# Lessons in AIDS Vaccine Development Learned from Studies of Equine Infectious, Anemia Virus Infection and Immunity

**DOI:** 10.3390/v5122963

**Published:** 2013-12-02

**Authors:** Jodi K. Craigo, Ronald C. Montelaro

**Affiliations:** Center for Vaccine Research, Department of Microbiology and Molecular Genetics, University of Pittsburgh, Pittsburgh, PA 15261, USA; E-Mail: craigoj@pitt.edu

**Keywords:** EIAV, lentivirus, pathogenesis, envelope diversity, vaccine development, immune maturation

## Abstract

Equine infectious anemia (EIA), identified in 1843 [1] as an infectious disease of horses and as a viral infection in 1904, remains a concern in veterinary medicine today. Equine infectious anemia virus (EIAV) has served as an animal model of HIV-1/AIDS research since the original identification of HIV. Similar to other lentiviruses, EIAV has a high propensity for genomic sequence and antigenic variation, principally in its envelope (Env) proteins. However, EIAV possesses a unique and dynamic disease presentation that has facilitated comprehensive analyses of the interactions between the evolving virus population, progressive host immune responses, and the definition of viral and host correlates of immune control and vaccine efficacy. Summarized here are key findings in EIAV that have provided important lessons toward understanding long term immune control of lentivirus infections and the parameters for development of an enduring broadly protective AIDS vaccine.

## 1. Introduction to EIAV

**Figure 1:**
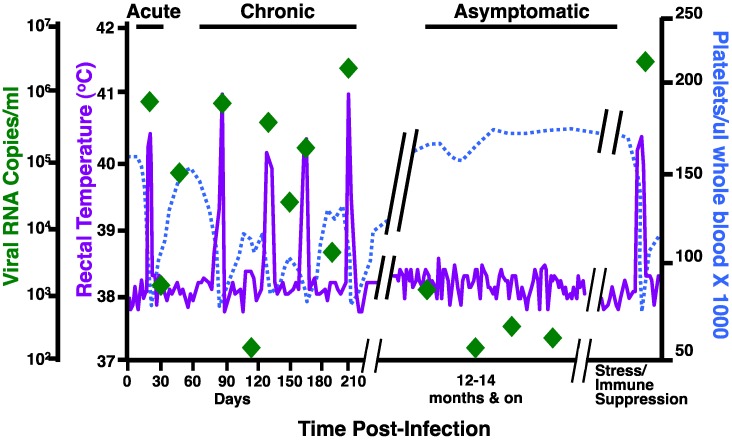
Profile of equine infectious anemia virus (EIAV) clinical disease. A schematic diagram representing a typical clinical profile over elapsed time associated with EIAV infection of equines indicating the characteristic stages of EIA disease. Febrile episodes are defined as rectal temperatures above 39 °C (103 °F), thrombocytopenia is defined as platelet levels below 105000/μL of blood, and a viremia of ≥10^5^ copies RNA/mL plasma.

EIAV, a lentivirus with strict macrophage tropism, causes a persistent infection in equines that results in a dynamic and chronic disease [2,3]. Transmission occurs primarily through the bite of blood-feeding horse flies, but can also happen parenterally through contaminated syringes in the field. Clinical EIA manifests in three basic phases: acute, chronic, and inapparent [3,4,5]. Waves of viremia induce acute and chronic disease episodes, which are defined markedly by clinical signs such as fever, anemia, thrombocytopenia, edema, lethargy, and various wasting symptoms. The most notable of the signs of disease are the concurrent development of fever and thrombocytopenia that are typically accompanied by viremia of at least 10^5^ copies RNA/mL plasma (Figure 1).

Disease cycling during the chronic stage continues for eight to twelve months post-infection. By one year post-infection, animals typically progress to life-long inapparent carriers with no outward clinical signs of disease. However, inapparent carriers still harbor low, stable levels of viral replication in monocyte-rich tissue reservoirs [3,6,7]. Stress or immune suppression of inapparent carriers can induce increases in viral replication and potentially a recrudescence of disease [3,8,9]. EIAV is unique amongst the majority of lentiviruses in that, despite incessant viral replication and associated rapid antigenic variation, greater than 90% of infected equids progress from a chronic disease state to an inapparent carrier stage, which is achieved by a strict immunologic control over virus replication [3]. EIAV infections of equines therefore serve as a unique animal model for the natural immunologic control of lentiviral replication and disease. Furthermore, inapparent carriers of EIAV have proven to be resistant to secondary infection with diverse viral strains, indicating the development of a high level of prophylactic immunity [2,3]. Thus, the EIAV system provides a valuable model for assessing lentiviral pathogenesis and persistence mechanisms, while identifying critical immune correlates of protection, and ascertaining the potential for developing effective prophylactic lentiviral vaccines. Interestingly, while EIAV provided the first natural model for immunologic control of lentiviral infection and disease, recent studies by Apetrei and colleagues have, from their survey of natural SIV infections, revealed a second model of natural immunologic control in African green monkey infected with SIVagm [10,11,12].

## 2. EIAV Antigenic Variation and Env Diversity: Lessons of Viral Evolution and Immune Evasion

Lentiviruses as a family, by the nature of their reverse transcriptase, exhibit rapidly evolving viral genomes. Natural and experimental EIAV infection results in a particularly vigorous disease process with pronounced demarcations of clinical disease (Figure 1) that offer a unique model with which to analyze the link between viral genetic variation, the origination of viral pathogenesis, and host immune responses to viral infection. All three clinical stages of EIA disease are associated with active viral replication. 

**Figure 2:**
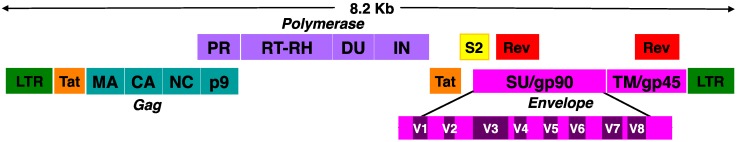
Schematic of EIAV viral genome. Organization of the EIAV genome indicating viral genes (italic print) and the respective proteins encoded by these genes (block print). V1-V8, indicates the defined Surface Unit, or gp90, variable regions.

The EIAV viral genome is characteristic of a complex retrovirus, but is the simplest and smallest of the described human and animal lentiviruses, possessing a mere three accessory proteins [2,3] (Figure 2). 

**Figure 3:**
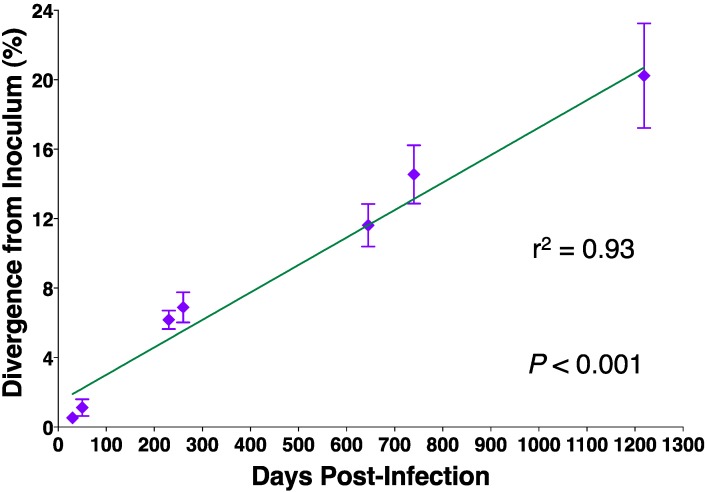
EIAV envelope divergence over time. Genetic distance calculations of amino acid sequence divergence of longitudinal isolate Envs from the inoculum Env sequence were calculated and plotted as a function of time. Linear regression (line) analysis revealed a significant linear relationship between the two variables (Figure modified from original published in Craigo *et al.* [26]).

Studies of EIAV genomic variation during persistent infection in experimentally infected horses and ponies have consistently identified changes predominantly in envelope sequences that alter viral antigenic properties, seemingly as a result of immune selection [9,13,14,15,16,17,18,19,20]. The prevalent site of EIAV Env variation during persistent infection is the surface unit or gp90 open reading frame. Analysis of the disposition of gp90 nucleotide and deduced amino acid variation defined distinct conserved and variable domains (Figure 2) [13,17,18] as observed with other animal and human lentiviruses [21,22,23,24,25]. The observed genomic alterations in the gp90 *env* gene, specifically in the identified variable regions, serve as a clear marker of viral population evolution. Examination of longitudinal gp90 variation during experimental infections revealed correlations between viral evolution, clinical disease, and immune control. These studies collectively demonstrated that a unique viral quasispecies appears with each febrile episode throughout acute and chronic EIA, with the dominant isolate appearing and disappearing with the cycling of disease [13,14,15,16,17,18,19]. The viral population of each disease episode, presumably now well recognized by the host immune system, is effectively and consistently eliminated and replaced with new quasispecies by the next febrile episode. This dynamic evolution is directly associated with the ebb and flow of viral replication that occurs during disease cycling associated with EIA. The level of divergence of the gp90 Env protein from the original viral inoculum and the population genomic diversity both increase throughout the course of longitudinal infection [26]. This significant, linear divergence from the original inoculum over time (Figure 3) affords the virus with an evolutionary advantage over the host immune system. 

Remarkably, a similar rate and extent of EIAV *env* evolution has been reported in progressor and nonprogressor ponies experimentally infected with EIAV, indicating that ongoing viral variation and population diversity occur in the absence of clinical indications and at relatively low levels of systemic virus replication [14]. Ultimately, viral evolution and associated increasing divergence and diversity of the quasispecies’ Env are responsible for the recurring nature of chronic EIA, which appears to be the result of the sequential evolution of EIAV antigenic variants that temporarily escape established immune surveillance. 

Comprehensive molecular characterization of EIAV Env variation within inapparent stage viral populations from experimentally infected ponies indicates that evolution of the viral quasispecies is continuous throughout all three clinical stages of disease, even with relatively low levels of detectable virus replication in the periphery or tissues [14]. These results suggest that even in the absence of detectable plasma virus, viral populations, likely in tissue reservoirs, continue to replicate and evolve, seeding the plasma with new quasispecies. Tissue reservoirs of lentiviral populations have been designated not only as active sites of replication, but also as reservoirs for latent lentiviral genomes [27,28]. The development of viral reservoirs contributes to the extraordinary level of persistence and in some instances pathogenesis associated with lentiviral infections [29,30]. Tissue reservoirs harbor stable pools of viral populations undergoing undetected low levels of replication or contain a latent viral population such as observed with HIV-1 found in resting memory CD4+ T cells [31,32]. In both cases, tissue reservoirs theoretically provide a stable archive of quasispecies reflecting the entire history of the viral infection. Thorough analyses of latent EIAV *env* sequences present during the inapparent stage of EIA disease was performed via chemical immune suppression of experimentally infected long-term inapparent carriers [20]. Viral populations from the periphery and tissues were examined through sequence analysis of the gp90. Upon comparison to previous viral populations, the immune suppression quasispecies appeared to be exclusively new viral population distinct from the quasispecies associated with prior acute and chronic EIA disease episodes [20]. 

**Figure 4:**
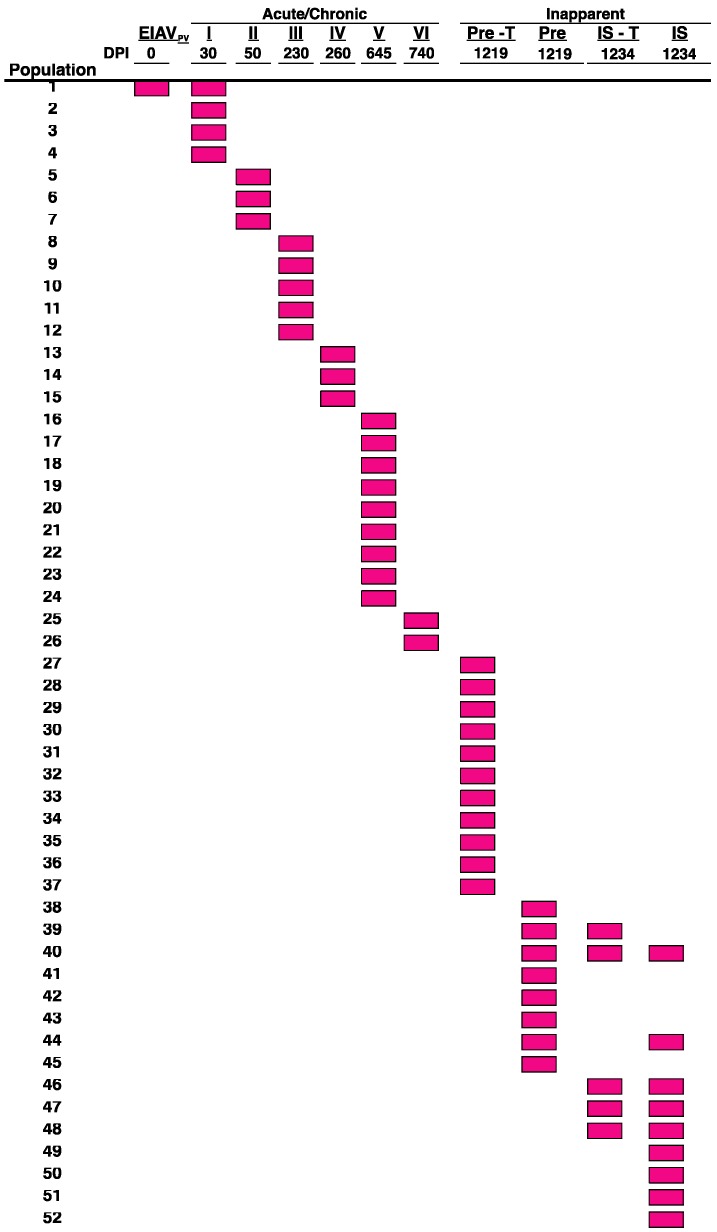
Temporal evolution of gp90 V3 envelope species demonstrates new species with each population. Temporal evolution of the gp90 Env V3 domain of longitudinal viral populations from an experimentally infected pony is represented graphically. Each distinct gp90 V3 region is depicted as a separate square, or population 1 through 52. Viral plasma species were determined for the acute and chronic stages from fever episodes I through VI, and from the inapparent stage prior to and during immune suppression in both plasma and tissues (lymph node, liver, and spleen). EIAV_PV_, lab strain inoculum; Pre-T, pre-immune suppression tissue; Pre, pre-immune suppression plasma; IS-T, immune suppression tissue; IS, immune suppression plasma (Figure modified from original in Craigo *et al*. [20]).

These observations indicate that the host immune system effectively eliminates a diverse array of antigenic variants, however, viral persistence is maintained by the relentless evolution of new Env populations from tissue reservoirs in response to ongoing immune pressures. 

Once again, studies of EIAV provided the first model to reveal the nature and role of rapid envelope antigenic variation in lentiviral persistence and pathogenesis, a theme now recognized as intrinsic to all lentiviral infections and a common challenge to vaccine development.

## 3. EIAV Diversity and the Host Immune Response: Lessons on Immune Maturation

The host immune system’s ability to control EIAV replication distinguishes this infection from the majority of the other lentiviruses and is the major determinant of disease in infected animals. Immune control of viral replication is perhaps best represented during the asymptomatic, or inapparent stage of infection, when equines are not actively shedding virus, yet their blood is still highly infectious to naïve equids [33]. EIAV-infected equines typically convert to seropositive in standard serological assays within three weeks post-infection. Humoral immune responses are predominantly against the viral Env glycoproteins, gp90 and gp45, and the major Gag core protein, p26. All current USDA-approved diagnostic assays for EIAV infection are based on the detection of antibody to the major core antigen. Remarkably, quantitative levels of EIAV Env-specific antibody remain relatively constant throughout the course of chronic EIA, including the inapparent stage of infection. However, beneath this steady state level of antibody responses, there is a characteristic evolution of EIAV-specific antibody populations that differ in qualitative properties, indicating a complex and lengthy maturation of humoral immunity to this viral infection.

Immune responses generated to evolving viral populations during acute and early chronic stages of EIA mediate significant pathogenesis in the presence of sufficient levels of EIAV antigenimia. Clearance of the initial infectious plasma viral quasispecies has been correlated with the emergence of EIAV-specific, major histocompatibility complex (MHC)-restricted, CD8 cytotoxic T lymphocytes (CTL) [34] and non-neutralizing Env-specific antibody [35,36]. Neutralizing antibody specific for the initial viral quasispecies emerges weeks after the primary viremic episode [37]. However, regardless of the identification of a clear relationship between viral diversity and host immune responses, well-defined correlates of immune protection towards vaccine development have not been definitively ascertained.

**Figure 5:**
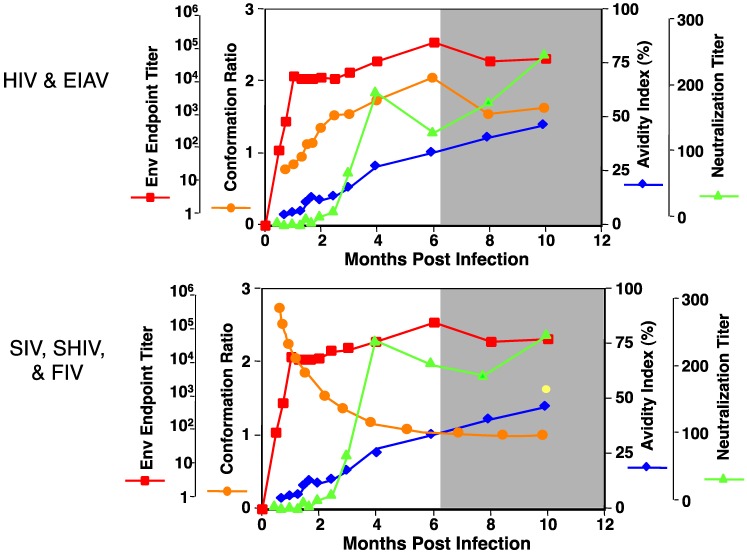
Schematic representation of lentiviral immune maturation. Development of mature immunity (grey-shaded box) occurs by 6–8 months post-infection. Red, reciprocal endpoint titer; Orange, conformational ratio; Blue, avidity index; Green, reciprocal neutralization titer (Figure modified from original in Montelaro *et al*. [39]).

Host humoral immune responses to EIAV require six to eight months to fully mature [7,38,39]. During this time frame, Env-specific antibody evolves progressively from a population characterized by low-avidity, non-neutralizing, and predominantly linear epitope specificity, to an antibody population with an avidity of moderate to high levels, neutralizing activity, and predominantly conformational epitope specificity (Figure 5). 

The complex and lengthy maturation of immune responses to EIAV infection has also been observed in sera from HIV-infected patients as well as experimental infections by SIV, SHIV, and FIV, indicating a common theme in lentivirus immunology. However, the progressive changes in qualitative antibody properties may differ among the different viral infections. For example, EIAV immune maturation of the humoral immune response resembles that identified in HIV, whereas SIV, SHIV, and FIV antibody maturation differ principally in the evolution of conformation ratios or the predominant recognition of linear/conformational epitopes [39]. Parallel serologic and genetic characterizations of EIAV quasispecies evolution have demonstrated a conclusive association between changes in viral neutralization specificity and variations in the sequence of the viral Env gp90 protein [13,14,17,18,40,41,42,43,44]. During the six to eight month immune maturation period, amino acid variations accumulated within the gp90 designated variable regions directly associate with distinct neutralization phenotypes [9,22,45]. The genotypic and phenotypic modifications observed within the gp90 variable regions contribute to viral persistence by temporarily evading established neutralizing antibody responses. Epitope mapping of gp90 variable regions when genomic diversity was maximal demonstrated that these sites of evolution are responsible for neutralization resistance and sensitivity, with identified domain exchanges between isolates reciprocally conferring either characteristic [16,45,46]. Therefore, the observed evolution in EIAV toward a neutralization-resistant phenotype appears to support the concept that Env variation results from immune selection, which increases antibody neutralization resistance, providing an important mechanism of persistence in the face of competent host immunity. 

Examination of the host cell-mediated immune response to EIAV reveals CD4 and CD8 MHC-restricted, EIAV-specific cytotoxic T-lymphocyte (CTL) activity apparent within three to four weeks post-infection, temporally correlating with the resolution of the primary viremia. After resolution of the initial acute episode, the breadth of EIAV-specific CTL activity differs greatly among experimentally infected ponies, ranging from readily detectable CTL to undetectable activity. The majority of the protective CTL responses appear to be directed towards Gag epitopes, although Env sequences have been loosely correlated with protective immunity [47,48]. Nevertheless, this correlation of envelope to CTL specificity has not been explored within the context of evolving sequences and overall diversity.

EIAV vaccine studies indicate a correlation between the extent of antibody maturation achieved by a particular immunization strategy and the potential for eliciting protective or enhancing vaccine immunity [39]. Although a true correlate of protection has not been identified for EIAV infection or vaccine-derived immunity, it is well established that the interplay between viral Env evolution and host immune responses is key to both immune escape as well as immune control of the lentivirus. The neutralizing competence of serum antibodies elicited to EIAV during chronic and inapparent stages of disease progressively increase, indicating an evolution of immune responses to the sequential generation of antigenic variants of virus. However, after the acute viremia resolves, there appears to be no single immune parameter that correlates with the resolution of further viremic episodes. Alternatively, immune control of EIAV infection during inapparent disease appears to rely on a complex combination of cellular and humoral immune responses to suppress viral replication. Nevertheless, studies of EIAV-specific immune response demonstrate that immune control is established only after the immune system has co-evolved with the virus to a fully mature state, six to eight months post-infection. These observations clearly indicate the requirement of lentiviral vaccines to achieve prolonged presentation of immunogens to drive immune maturation and to achieve protective status.

## 4. Env Diversity and EIAV Vaccines: Lessons on Efficacy and Protection

Lentiviral Env diversity has a tremendous effect on the host’s ability to control viral infection and disease. As the viral envelope protein is a primary source of antigenicity and interacts with the host immune system to a much larger extent than any other viral protein, it is often the main target in vaccine development. Thus, Env diversity has a remarkable impact on the development of an effective vaccine for lentiviruses in general as well as specifically for control of worldwide EIAV infections. 

Diverse experimental EIAV vaccine strategies have been evaluated for their ability to achieve mature protective immune responses. These exploratory vaccine trials include various live-attenuated (attenuated) virus strains, inactivated whole virus candidates, DNA, and particulate immunogens, viral or recombinant envelope protein subunit immunogens, and Gag subunit immunogens [26,49,50,51,52,53,54,55,56,57,58,59]. The cumulative results of these studies reveal an extraordinary spectrum of vaccine efficacy, ranging from decidedly type-specific protection to severe enhancement of viral replication and disease in immunized horses and ponies challenged with reference virus strains. The observations suggest vaccine immune responses can be duplicitous, mediating protection or enhancement upon virus exposure. The results also reveal important determinants of vaccine efficacy. One of the early observations noted a general association between the extent of envelope-specific antibody maturation achieved by experimental immunization and protective efficacy. The studies demonstrated a critical importance of the mode of Env antigen presentation in affecting immunogenicity and the specificity and protective efficacy of vaccine immunity. Primarily, it is essential that Env-based vaccines provide antigen presentations that elicit mature protective immunity and avoid immature non-protective and potentially enhancing immunity. 

The cumulative data on infection and candidate vaccine trials indicate that viral Env protein is indeed a primary determinant of vaccine efficacy and that the natural variation observed in Env quasispecies represents a major challenge to vaccine development. A significant, inverse, linear correlation between vaccine efficacy and increasing divergence of the challenge virus gp90 compared to the vaccine virus gp90 protein has been identified utilizing EIAV attenuated vaccine studies [54]. The results demonstrated approximately 100% protection from disease of immunized horses after challenge by virus with a homologous gp90, but only 40% protection against challenge by virus with an Env gp90 that was 13% divergent from the vaccine strain. The calculated linear relationship predicted a complete lack of protection of immunized horses from disease upon challenge with a virus gp90 that is 23% divergent from the vaccine strain. Additionally, analysis of attenuated vaccine strain day of challenge Env evolution described a significant relationship between protection from disease and divergence of the attenuated strain gp90, not overall attenuated strain gp90 population diversity [26]. Hence, the EIAV model established the first conclusive relationship between defined natural lentivirus Env variation and vaccine efficacy, and revealed that an efficacious immunogen should represent antigen divergence and not merely a diverse population of Env proteins. Furthermore, observations from EIAV vaccine trials have important implications for immunogen design towards the development of vaccines that produce enduring broadly protective immunity to the diverse quasispecies expected in circulating HIV-1 populations in different geographical areas. 

## 5. Conclusions

Studies of EIAV pathogenesis, host immune response, and vaccine efficacy have provided many invaluable lessons for HIV/AIDS research. EIAV, like HIV, possesses an extremely variable genome with the majority of genetic mutations centralized within the Env protein. Env evolution and diversity have profound effects on host immune control of the infection and disease, as well as the development of an effective vaccine. The prominent demarcation of viremic episodes as well as the unique ability of the host to naturally achieve immunological control over disease made EIAV studies highly relevant to understanding basic principles of lentivirus pathogenesis. Furthermore, EIAV research has promoted fundamental understanding of the mechanisms by which Env variation circumvents vaccine immunity and informed the development of novel immunization strategies to maximize effective immune recognition of variant Env species. 
